# Pelvic Lipomatosis Causing Renal Failure

**DOI:** 10.5334/jbr-btr.1072

**Published:** 2016-04-08

**Authors:** Isabeau Hermie, Laurens Hermie, Kenneth Coenegrachts

**Affiliations:** 1 AZ St-Jan Brugge-Oostende, BE; 2 UZ Gent, BE

**Keywords:** fatty infiltration of the abdomen, abdominal mass, pelvic lipomatosis, CT

## Observation

A 34-year-old Cape Verdean male with terminal chronic renal failure of unknown etiology since two years was referred to the urology department for a second opinion after finding an infiltrative mass on routine renal ultrasound. The patient was nonobese and otherwise asymptomatic. Clinical examination revealed a large palpable mass in the pelvis. Laboratory examination showed highly elevated blood levels of creatinine (10.98 mg/dL (nl: 0.67–1.17 mg/dL)) and urea (123 mg/dL (nl: 16.6–48.5 mg/dL)).

**Figure 1:**
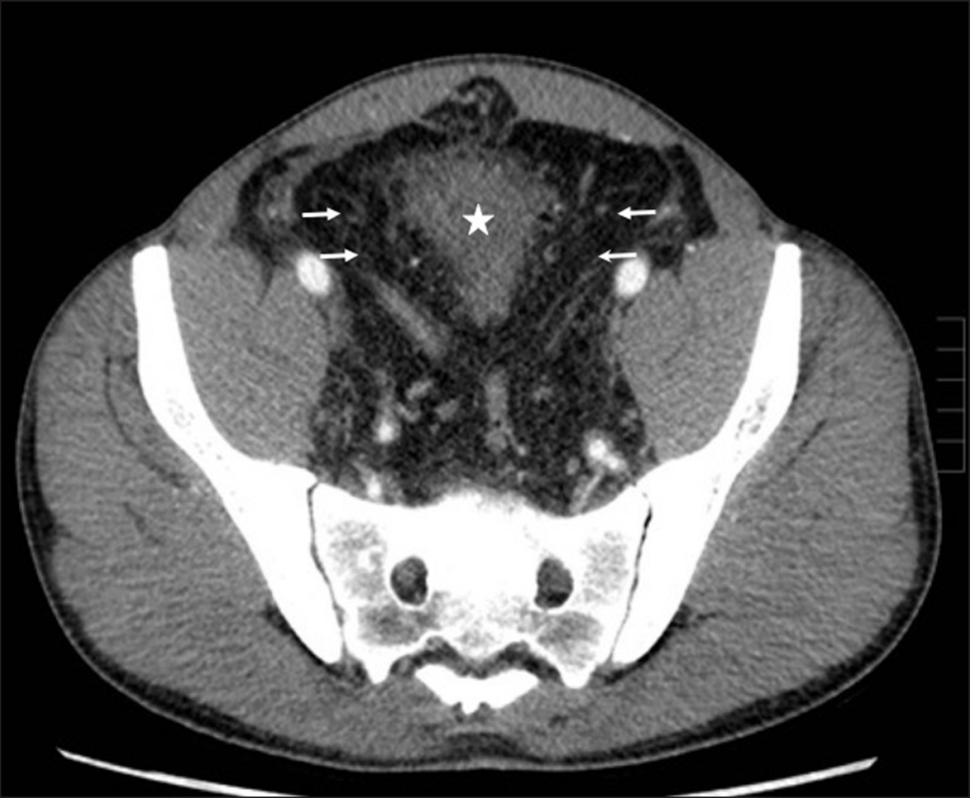
Contrast enhanced computed tomography. Axial image showing the presence of uncapsuled hypodense fat in the pelvis (arrows), causing extrinsic pressure of the bladder (white star) and the rectum.

**Figure 2:**
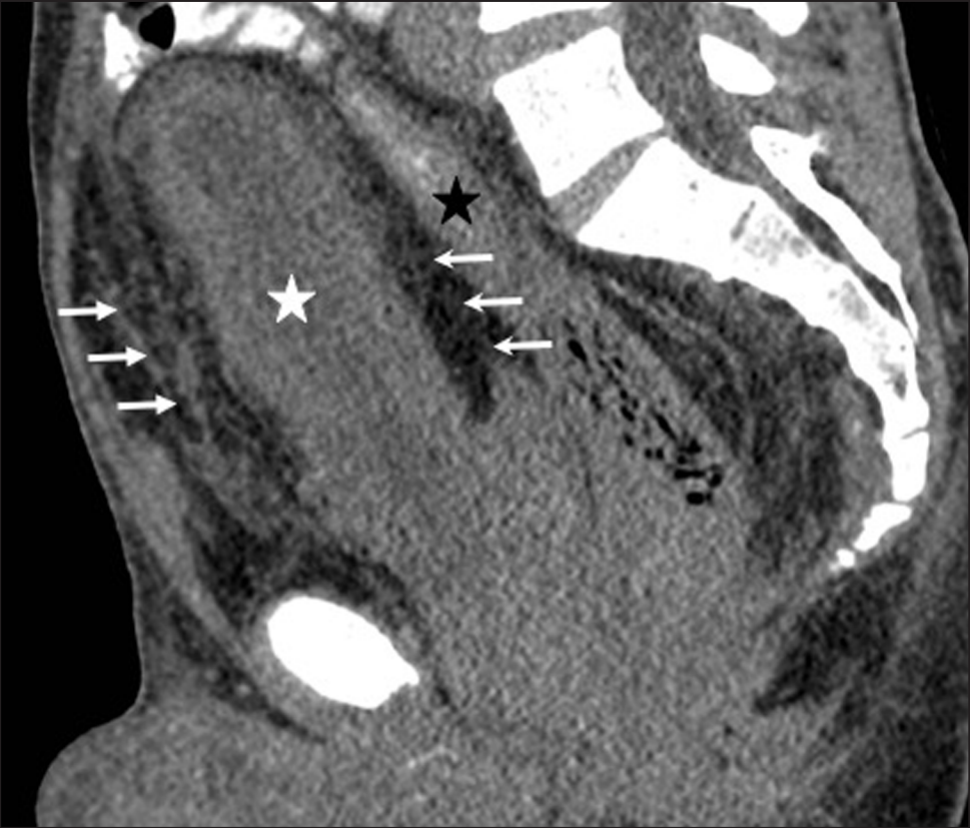
Contrast enhanced computed tomography. Sagittal image displaying the anatomical deformity of the bladder (white star) and the rectum (black star) caused by the deposition of hypodense fatty tissue (arrows).

**Figure 3:**
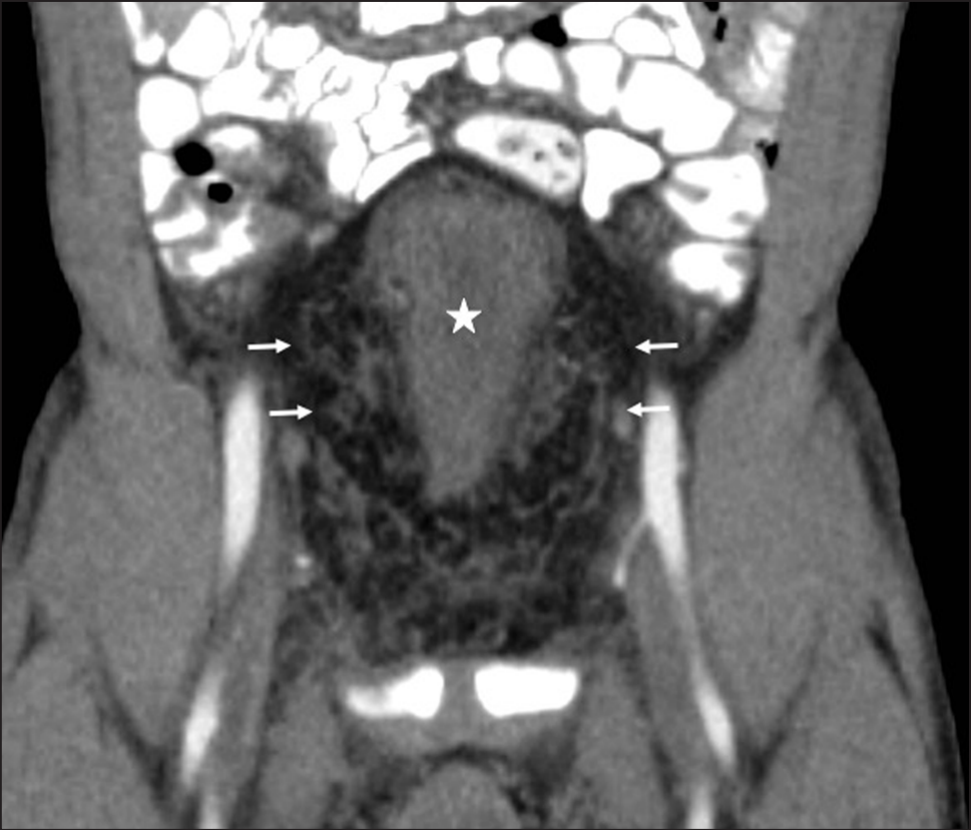
Contrast-enhanced computed tomography. Coronal imaging showing a ‘pear’-shaped bladder (white star) surrounded by hypodense fatty tissue (arrows).

We decided to perform a contrast-enhanced CT scan for further evaluation of the mass. The application of contrast agents in this patient with renal failure was permitted because these contrast agents were washed out by dialysis afterwards. The CT examination showed a large pelvic infiltration by fatty tissue with typical attenuation (–40 to –100 Hounsfield units) (Figure 1, arrows) and bilateral small kidneys with atrophy and hydroureteronephrosis. The fatty tissue caused extrinsic compression of the bladder (Figures 1,2, and 3, white star) and rectum (Figure 2, black star) resulting in morphological deformity. The findings led to the diagnosis of pelvic lipomatosis with secondary chronic renal failure.

## Comment

Pelvic lipomatosis is an uncommon disease that was first described by Engels et al. in 1959 and is characterized by overgrowth of nonencapsulated, nonmalignant but infiltrative adipose tissue [1]. It is usually symmetric and limited to the pelvis, with extension in the retroperitoneum in rare cases.

It presents a broad range of symptoms, usually caused by compression of pelvic structures. The urinary tract (pollakiuria, dysuria, nocturia, and hematuria); the lower intestinal tract (constipation, tenesmus, rectal bleeding, and ribbon-like stool with mucus); and the vascular system (edema of lower extremities) can be compressed. At physical examination, one may observe pain at abdominal palpation, presence of a palpable mass in the hypogastric region, urinary retention, elevation of the prostate on digital rectal examination, lower limb edema, and arterial hypertension. Late complications are obstructive renal failure, proliferative cystitis, and bladder adenocarcinoma.

The etiology of pelvic lipomatosis is unknown. Some authors have raised the hypothesis that this disease is a manifestation of generalized obesity; other authors have suggested it is a response to recurrent urological infections. The disease is more prevalent in males and African Americans.

Imaging studies such as CT or MRI are crucial in the diagnosis of pelvic lipomatosis. Diagnosis is based on the distinct absorption coefficient of the intrapelvic fatty tissue. The pelvis seems congested because of excessive amounts of symmetrically spread fat. Soft-tissue planes of the pelvis are preserved. There is no truly soft-tissue mass present, and after the administration of a contrast agent, the fat does not enhance. The urinary bladder is extrinsically compressed, vertically stretched out, and raised from the pelvic floor. On ultrasound, there is hyperechogenic fat tissue surrounding the pear-shaped bladder and sigmoid.

Treatment options are limited. Dietary management, antimicrobial agents, corticoids, and radiotherapy have not demonstrated efficacity. Complete surgical eradication of the adherent fatty tissue is difficult, and the clinical effect of this intervention is uncertain.

The possibility of making a specific diagnosis of pelvic lipomatosis without an invasive technique is useful. Therefore, one should also think about the possibility of pelvic lipomatosis in a patient with chronic renal failure of unknown etiology with an increased amount of adipose tissue in the pelvis in the absence of a truly mass lesion.

## Competing Interests

The authors declare that they have no competing interests.
